# Testing of novel spectral device sensor in swine model of airway obstruction

**DOI:** 10.14814/phy2.14246

**Published:** 2019-10-06

**Authors:** Megan B. Blackburn, Corinne D. Nawn, Kathy L. Ryan

**Affiliations:** ^1^ Tactical and En Route Care Department U.S. Army Institute of Surgical Research JBSA Fort Sam Houston Texas; ^2^ Department of Biomedical Engineering University of Texas at San Antonio San Antonio Texas

**Keywords:** Airway, Respiration, Spectral Reflectance, Trauma

## Abstract

Loss of a patent airway is a significant cause of prehospital death. Endotracheal intubation is the gold standard of care but has a high rate of failure and complications, making development of new devices vital. We previously showed that tracheal tissue has a unique spectral profile which could be utilized to confirm correct airway device placement. Therefore, the goals of this study were twofold: 1‐ to develop an airway obstruction model and 2‐ use that model to assess how airway compromise affects tissue reflectance. Female swine were anesthetized, intubated, and instrumented. Pigs were allowed to breathe spontaneously and underwent either slow‐ or rapid‐onset obstruction until a real‐time pulse oximeter reading of ≤50%. At baseline, 25%, 50%, 75%, and 100% obstruction, a fiber‐optic reflection probe was inserted into the trachea and esophagus to capture reflectance spectra. Both slow‐ and rapid‐onset obstruction significantly decreased arterial oxygen concentration (sO_2_) and increased partial pressure of CO_2_ (pCO_2_). The presence of the tracheal‐defining spectral profile was confirmed and remained consistent despite changes in sO_2_ and pCO_2_. This study validated a model of slow‐ and rapid‐airway obstruction that results in significant hypoxia and hypercapnia. This is valuable for future testing of airway device components that may improve airway management. Additionally, our data support the ability of spectral reflectance to differentiate between tracheal and esophageal tissues in the presence of a clinical condition that decreases oxygen saturation.

## Introduction

Trauma is a leading cause of death in the civilian population (Prevention CfDCa, 2017). In trauma patients, airway management is the highest priority as demarcated by the ABCs of Advanced Trauma and Life Support guidelines for initial assessment (airway, breathing, and circulation) (Support ACoSCoTSoATL, 1989). Severe consequences occur from lack of a patent airway, making airway compromise a significant cause of trauma‐related death in both the civilian and military populations (Evans et al., 2010; Eastridge et al., 2012), indeed, it is the second leading cause of potentially survivable death on the battlefield (Eastridge et al., 2012). Clinically, endotracheal intubation (ETI) is the gold standard for nonsurgical airway management and is frequently performed in trauma patients in environments ranging from life‐threatening prehospital situations such as cardiac arrest or trauma (ambulance, battlefield, etc.) to the emergency and operating rooms.

ETI has been the definitive, nonsurgical approach to airway management since the inception of the laryngoscope in the late 1800s. The intricate procedure involves the provider using a laryngoscope to apply forceful leverage on the upper airway structures in order to obtain visualization of the vocal cords and pass the endotracheal tube through the glottic opening and into the trachea, thereby securing a patent pathway for respiration. Consequently, ETI is a difficult skill to acquire and maintain and evidence suggests that obtaining a 90% proficiency rate requires experience with 75 intubation cases (Je et al., 2015), a number of cases which is often unattainable for many prehospital providers. Without sufficient training and experience, significant complications, such as hypoxia, hypotension, aspiration, and cardiac arrest, may occur. Additionally, first pass success is vital as multiple attempts at ETI significantly increase the risk of adverse events (Sakles et al., 2013).

One of the most life‐threatening complications encountered during intubation procedures is esophageal intubation. This procedural error results in the endotracheal tube being placed into the esophagus, which lays just posterior to the trachea, and air is then passed into the stomach rather than the lungs. Unrecognized esophageal intubations have been reported more frequently in the prehospital space and can ultimately lead to death (Timmermann et al., 2007; Lockey et al., 2013). In order to prevent this, the 2010 Advanced Cardiac Life Support guidelines recommends a confirmatory procedure be performed after every ETI to ensure correct placement in the trachea (Neumar et al., 2010). The preeminent standard for correct tube placement is visualization of the tube passing through the vocal cords and observation of the chest rising and falling. However, visualization may be compromised in “difficult airways” in which there is trauma to the face and/or neck. In instances where visualization is not possible, auscultation may be performed to listen for bilateral breath sounds (though this may be difficult in the prehospital environment) or expired CO_2_ monitoring can be done using colorimetry and/or capnography. Colorimetry uses color‐changing paper that responds to varying levels of CO_2_ in expired breath but this method is subjective and not continuous (Johnson et al., 2008). Capnography is a continuous waveform measurement of end‐tidal CO_2_ often used in the clinical setting and proven to provide strong benefits in regards to confirmation of proper endotracheal tube placement (Rabitsch et al., 2004) and continuous patient monitoring (Rabitsch et al., 2004; Cook, 2018). Despite that, many emergency departments do not employ its usage and monitors are not carried by first responders on the battlefield (Deiorio, 2005). Furthermore, capnography may not always be a reliable indicator of correct ETI as end‐tidal CO_2_ can be influenced by low cardiac output states or lung pathology (Shibutani et al., 1994). Consequently, there exists a need for a nonvisual detection mechanism for continuous confirmation of proper endotracheal tube placement that can rapidly give the user feedback throughout the process of intubation and subsequently in cases of patent airway loss or imminent airway compromise. This is especially important considering that, even when placed correctly, endotracheal tubes can become unknowingly dislodged during patient transport.

Previous studies performed by our laboratory showed that tracheal and esophageal tissues reflect white light differently and produce significantly different spectral responses in *ex vivo* and *in vivo* settings (Nawn et al., 2016; Nawn et al., 2019). This novel methodology of utilizing spectral reflectance may prove to be a clinically relevant technology that could be leveraged as a confirmatory method for correct endotracheal tube placement. Given the frequency of death as the result of airway compromise in trauma patients and high rate of intubation complications in the prehospital environment, such a technology could fill the gap of accurate, nonvisual confirmation of tracheal placement suitable for prehospital and battlefield environments. Currently, there is no animal model of airway obstruction in which to test novel device components such as spectral reflectance, thereby limiting the ability to advance airway management. The purposes of this study were to first develop a model of hypoxia and hypercapnia in swine to simulate both gradually developing and sudden‐onset airway compromise, and then use that model to assess the spectral reflectance of tracheal and esophageal tissues *in vivo* under normoxic, hypoxic, and hypercapnic conditions.

## Materials and Methods

This study was approved by the Institutional Animal Care and Use Committee of the US Army Institute of Surgical Research, San Antonio, TX. Research was conducted in compliance with the Animal Welfare Act, the implementing Animal Welfare Regulations and the principles of the Guide for the Care and Use of Laboratory Animals, National Research Council. The facility where this research was conducted is fully accredited by AAALAC International.

### Animal Preparation and Instrumentation

A total of 16 animals were used in this study, 8 to establish the model of airway obstruction and 8 to determine spectral reflectance values. Female Yorkshire pigs (approximately 40 kg) were induced with an intramuscular injection of tiletamine‐zolazepam (Telazol, 4–6 mg/kg), intubated with cuffed ET tubes (8 mm internal diameter) in the trachea and esophagus, and secured on the table in a supine position. During instrumentation, animals were mechanically ventilated with positive pressure 30% O_2_ at 8–10 mL/kg per breath and 8–12 breaths per minute to maintain an end‐tidal pCO_2_ of 45 ± 5 mmHg. Positive end expiratory pressure was set to 5 cmH_2_O. Anesthesia was provided with 1–3% isoflurane. Maintenance fluid (lactated Ringer’s solution at 3–5 mL/kg per h) was administered through an ear vein catheter and urine output was collected via a Foley catheter. Body temperature was monitored via a rectal probe and maintained at 37–39^°^C. A pulse oximeter (Nellcor) was attached to the tongue or cheek for measuring peripheral oxygen saturation levels (SpO_2_) and ECG leads attached for heart rate (HR). The right carotid artery was cannulated for continuous recording of blood pressure (BP) and the left femoral artery was cannulated for blood sampling. All blood analysis was performed via iStat. Once instrumented, isoflurane was replaced with continuous intravenous infusion of midazolam (0.6–2.5 mg/kg per h), propofol (3–6 mg/kg per h), and buprenorphine (2–10 µg/kg per h) and animals were allowed to breathe room air spontaneously. Depth of anesthesia was monitored based on breathing pattern, hemodynamic variables, jaw tone, and lack of a withdrawal reflex in response to toe pinch.

### Airway obstruction

**Figure 1:**
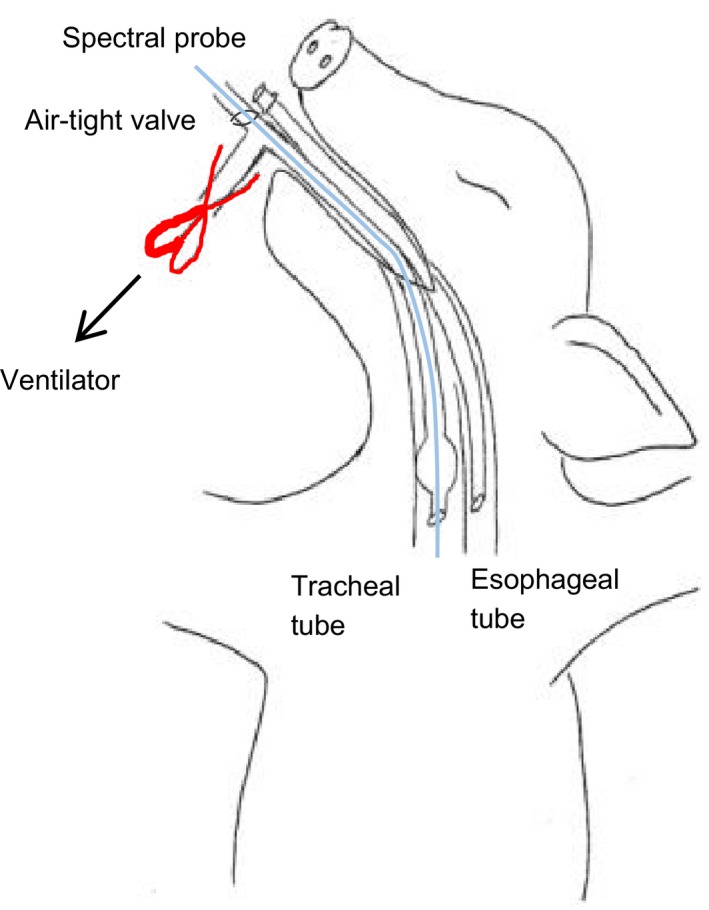
Experimental set‐up utilizing a specialized valve to allow for air‐tight passage of probes into the trachea.

The goal of the protocol was to mimic the physiological conditions of both impending airway compromise and complete loss of a patent airway. Thus, each animal underwent both slow and rapid obstruction, with the order of these perturbations alternated across animals. To impede air flow, a large thoracic hemostatic clamp was placed on the ventilation tubing connected to the proximal end of the endotracheal tube (Fig. 1). A valve provided an airtight seal but still allowed the passing of a probe into the trachea. Slow‐onset airway obstruction consisted of four levels of obstruction (25%, 50%, 75%, and 100%) obtained by slowly ratcheting the hemostatic clamp until fully closed and maintaining each level for 8 min. Each level of obstruction was approximated based on the degree to which the hemostatic clamp was ratcheted closed. Here 100% obstruction was maintained until oxygen saturation fell to ≤50% as measured by pulse oximeter. Rapid‐onset airway obstruction was obtained by immediately and completely clamping the valve via the large thoracic hemostatic clamp. Once the oxygen supply was completely obstructed, it was not released until oxygen saturation fell to ≤50% as measured by pulse oximeter.

### Spectral reflectance measurements

Reflectance spectra were captured using a fiber optic reflection probe (Gulf Photonics) connected to a compact spectrometer and halogen white light source (Ocean Optics) as described previously (Nawn et al., 2016; Nawn et al., 2019). Briefly, the customized right‐angle fiber optic probe was configured to emit the white‐light, illuminating the surrounding tissue, and to capture the light reflected from the luminal tissue. SpecSoft software was used to display the spectra in real time and store the captures for post‐analysis. To account for background interference and the imperfections of the white light source, dark and white reference spectra were collected and stored before each experiment. Figure 1 shows the instrumentation of the ET tubes in the trachea and esophagus alongside the relative placement of the fiber optic probe through the air‐tight valve, into the trachea. At the start of each experiment, a bronchoscope (Olympus) or Ambu aScope™ was inserted through the ET tube to take images and measure depth to varying positions: just distal to the ET tube, at the level of the accessory lobe, and just proximal to the carina. The spectral probe was then positioned to capture the reflectance spectra in real‐time. Five snapshots were taken at the three corresponding levels in both the trachea and esophagus and the probe was cleaned with an isopropanol surface disinfectant wipe between captures of each tissue type.

### Experimental design

A 15‐min baseline of hemodynamic and respiratory parameters was recorded, followed by slow‐ or rapid‐onset airway obstruction. Once oxygen saturation reached 50%, the hemostatic clamp was released and the animal recovered for 30 min before undergoing the second obstruction. Spectral measurements were taken at all three levels (just distal to the ET tube, at the level of the accessory lobe, and just proximal to the carina) at baseline, each level of obstruction during slow‐onset, and once fully obstructed during rapid‐onset. Arterial blood samples were taken at each obstruction level at the same time as spectral measurements for blood gas analysis.

### Data analysis

The reflectance, absorbance, and amplitude spectra were collected through the SpecSoft Software, stored as text files, and imported into MATLAB for post‐analysis. Each capture was annotated numerically, according to the pig, and classified as tracheal or esophageal based on the relevant tissue type. All tracheal and esophageal spectra were first plotted separately to inspect for signal fidelity or any erroneous captures. Previous work identified 500 nm to 650 nm as the range of interest, and therefore all spectra were cropped to focus on that region. The amplitude values at 543 nm, 561 nm, and 578 nm were extracted for each capture (wavelengths chosen based on previous studies; Rabitsch et al., 2004, Prevention CfDCa, 2017) and plotted to visualize the distribution. The Wilcoxon Signed Rank Sum test was used in comparisons between tissue types.

All hemodynamic data are expressed as mean ± SEM. For all variables, 30 sec segments at each time point were compared to a 5‐min baseline period measurement. Data were analyzed by two‐way ANOVA with repeated measures from baseline to full obstruction. A value of *P* < 0.05 identified significant group or time effects. Multiple comparisons were conducted using the Holm–Sidak method (GraphPad Prism).

## Results

### Models of hypoxia and hypercapnia

**Figure 2:**
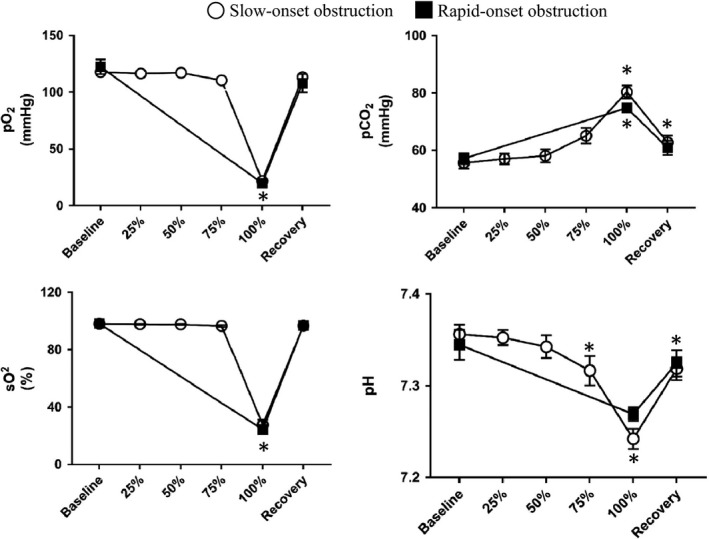
Summary data of the effects of slow‐ and rapid‐onset airway obstruction on pO_2_, pCO_2_, sO_2_, and pH (*n* = 16). Both slow‐ and rapid‐onset obstruction significantly decreased pO_2_, SO_2_, and pH while increasing pCO_2_. Values are mean ± SEM *Significant difference versus baseline (*P* < 0.05).

**Figure 3:**
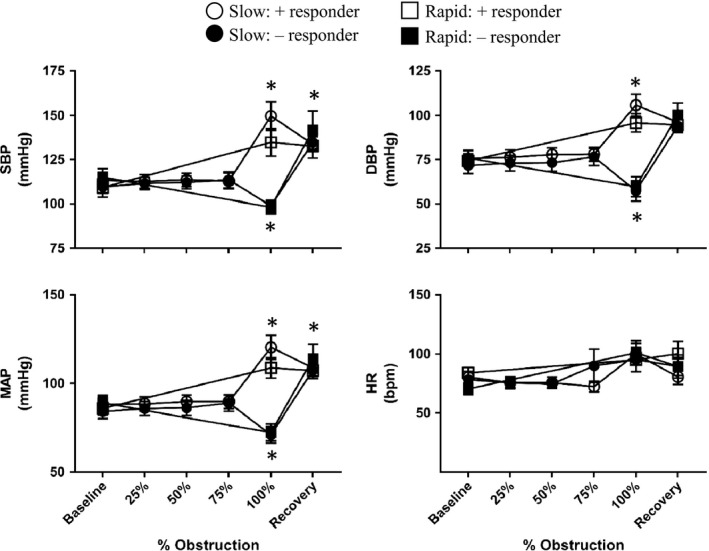
Summary data of the effects of slow‐ and rapid‐onset airway obstruction in positive (*n* = 9) and negative (*n* = 7) hemodynamic responders. SBP, DBP, and MAP were significantly increased and decreased in positive and negative responders, respectively, once 100% obstruction was reached. HR was not significantly altered in any group. Values are mean ± SEM *Significant difference versus baseline (*P* < 0.05).

Slow‐ and rapid‐onset obstruction induced both hypoxia and hypercapnia as desired. Obstruction significantly decreased oxygen partial pressure (pO_2_), arterial oxygen saturation (sO_2_), and pH and increased CO_2_ partial pressure (pCO_2_) (Fig. 2). pO_2_ and sO_2_ were not significantly altered during slow‐onset obstruction until full obstruction was reached. Once complete obstruction was achieved, there was no significant difference in sO_2_ or pCO_2_ between those that underwent slow‐ versus rapid‐obstruction. Interestingly, opposed to our expectation that arterial blood pressure would increase significantly in response to obstruction, animals responded in one of two ways: one group had a significant elevation in MAP (*n* = 9, positive responders), while the other had a significant decrease (*n* = 7, negative responders) (Fig. 3). HR was not significantly different compared to baseline at any time point between the four groups and the amount of time it took to reach an spO_2_ < 50% was not significantly different between positive and negative responders.

### Spectral reflectance

**Figure 4:**
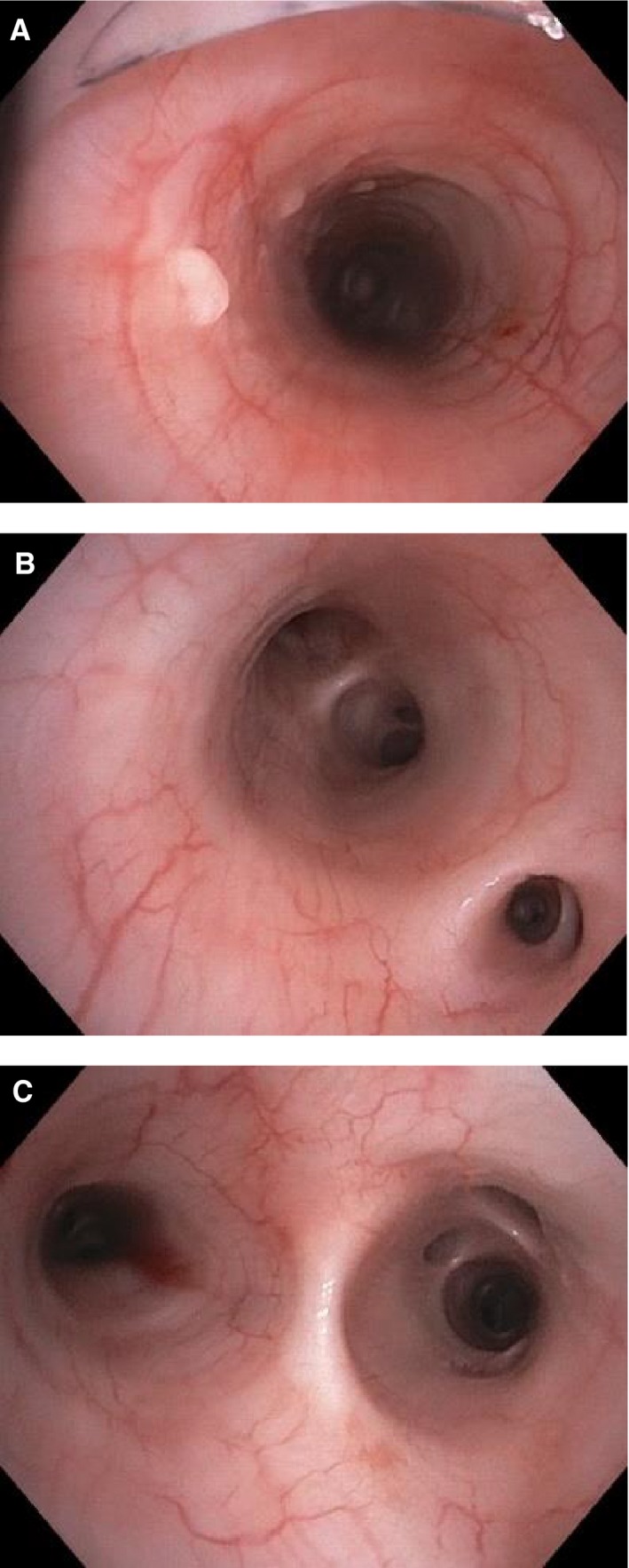
Representative images taken at the (A) distal edge of the endotracheal tube, (B) level of the accessory lobe, and (C) proximal to the carina, wherein spectral measurements were taken.

Spectral measurements were taken on eight pigs, in three locations: just distal to the ET tube, at the level of the accessory lobe, and just proximal to the carina. In order to verify location, bronchoscopy images were taken at the start of each experiment (Fig. 4) and depth measurements used for spectral captures. The first objective was to determine if location within the trachea or esophagus significantly altered the spectral profile of the tissue. Table 1 shows the baseline tracheal and esophageal reflectance values at all three locations. There was no significant difference in reflectance at 543 nm, 561 nm, or 578 nm between any of the locations, and therefore all reflectance values at each depth were averaged together for further analysis.

**Table 1:** Baseline tracheal and esophageal reflectance values just distal to the endotracheal tube (upper), at the level of the accessory lobe (middle), and proximal to the carina (lower).

Wavelength	Upper Median (IQR)	Middle Median (IQR)	Lower Median (IQR)	*P*‐value
Tracheal Reflectance				
R543	0.985 (0.006)	0.986 (0.005)	0.985 (0.004)	0.692
R561	0.993 (0.005)	0.993 (0.003)	0.992 (0.003)	0.843
R578	0.987 (0.006)	0.987 (0.005)	0.987 (0.007)	0.932
Esophageal Reflectance				
R543	0.993 (0.003)	0.994 (0.003)	0.993 (0.004)	0.658
R561	0.996 (0.003)	0.996 (0.003)	0.995 (0.004)	0.563
R578	0.995 (0.004)	0.995 (0.004)	0.994 (0.007)	0.478

**Figure 5:**
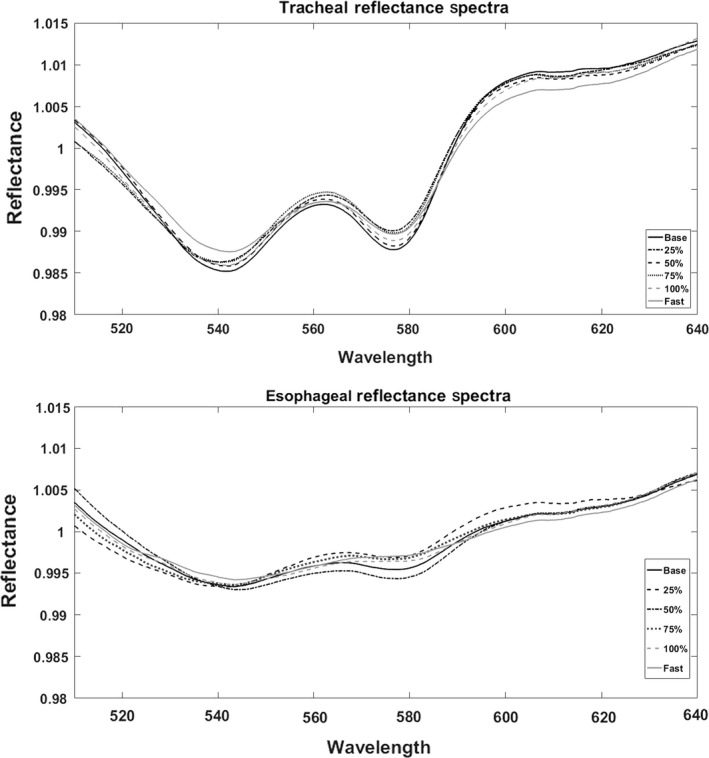
Averaged tracheal (top) and esophageal (bottom) spectra during baseline, 25%, 50%, 75%, and 100% obstruction during both slow‐ and rapid‐onset (*n* = 8).

**Figure 6:**
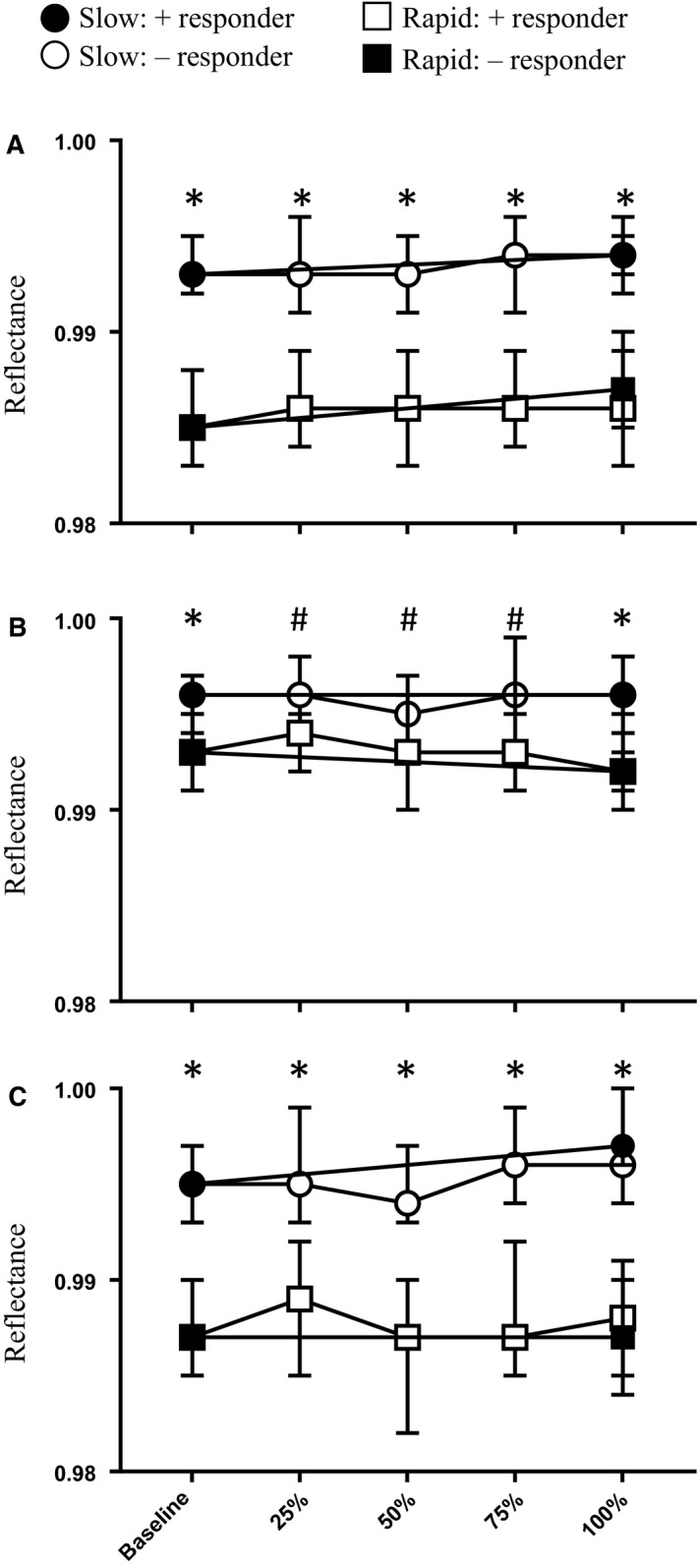
Summary data of tracheal and esophageal reflectance values during both slow‐ and rapid‐onset airway obstruction at (A) 543 nm, (B) 561 nm, and (C) 578 nm. Values are median and IQR, *P < 0.001, #*P* < 0.05 for differences between tracheal and esophageal reflectance at each level of obstruction.

The main objective of this study was to determine spectral reflectance of the trachea and esophagus in the presence of hypoxia and hypercapnia. Figure 5 shows the averaged spectral profile of the trachea (top) and esophagus (bottom) for each level of obstruction. Qualitatively, the profiles did not differ. Tracheal spectra generally exhibited the same peak as previously described (Nawn et al., 2016; Nawn et al., 2019), with minima at 543 nm and 578 nm and a maximum at 561 nm. The esophageal profile also displayed a slight peak at 561 nm but the peak is much less prominent. A quantitative comparison of the reflectance values for both tracheal and esophageal measurements at each wavelength during both slow‐ and rapid‐obstruction are shown in Figure 6. Neither tracheal nor esophageal reflectance was altered over time during obstruction, to include baseline and full obstruction. Additionally, reflectance values at all three wavelengths differed significantly between trachea and esophagus, and this difference was maintained during hypoxia and hypercapnia.

## Discussion

Despite the high complication rates and difficulty in maintaining procedural efficacy, ETI is a procedure that has largely been unchanged in the last century in regards to performance by first responders. In order to progress the airway management field via device development and testing, an innovative *in vivo* model of airway compromise is necessary to scientifically evaluate devices. The goal of this study was twofold: first to develop a large animal model of airway obstruction then to use this model to test a novel method of airway sensing, namely spectral reflectance. Based on our previous studies (Nawn et al., 2016; Nawn et al., 2019), we hypothesized that tracheal tissue would have a distinct spectral profile that would persist despite location within the trachea, hypoxia, or hypercapnia; this hypothesis was confirmed.

Our first goal was to develop a model of airway compromise wherein we could elicit hypoxia and hypercapnia while still having access to the trachea and esophagus for the purpose of developing a relevant model in which innovative technologies could be evaluated. To do this we utilized a valve (as shown in Fig. 1) which created an air‐tight seal while still allowing the passage of probes. Given our interest in trauma and battlefield‐relevant research, we aimed to develop both a slow‐ and rapid‐onset obstruction, we were able to simulate and compare gradually developing airway compromise, similar to what may occur as the result of tissue swelling, versus that of rapid asphyxia, similar to what may occur as the result of sudden obstruction due to maxillofacial trauma. Based on previous model development experiments, we selected 50% oxygen saturation as a cut‐off which was significant enough to elicit hemodynamic changes while still allowing the animals to fully recover from the insult. As expected, both slow‐ and rapid‐onset obstruction decreased oxygen content and pH while increasing CO_2_ partial pressure. Interestingly, few differences were seen during slow‐onset obstruction until 100% obstruction was reached, with only pCO_2_ and pH being significantly altered before full obstruction.

We hypothesized that both slow‐ and rapid‐onset obstruction would increase MAP (via sympathoexcitation) (O'Donnell et al., 1996; Ferreira et al., 2018). Indeed, this occurred in 56% (9/16) of animals, while in 44% (7/16) MAP was decreased in response to airway obstruction. The reasons for this dichotomy are unknown. The majority of studies examining hemodynamic responses to airway compromise have been done in the context of sleep apnea with repetitive, acute bouts of hypoxemia or hypercapnia in rodents. Varying results are found in those instances, with both increases and decreases in MAP observed. It is important to note that animals were under anesthesia, and while every effort was made to ensure that animals were under similar states of anesthesia, it is possible that disparate amounts caused varying responses to obstruction. Additionally, we did not record sympathetic nerve activity in these animals, and therefore cannot speculate on the autonomic response during obstruction in our study.

Current guidelines recommend ETI for trauma patients with a Glascow Coma Score of 8 or less or sO_2_ of <90% (Mayglothling et al., 2012). Additionally, it is also recommended that a confirmatory procedure be performed after every ETI to verify tracheal placement (Neumar et al., 2010). If spectral reflectance is to be used for placement confirmation, the probe could be placed on the tip of either a bougie or endotracheal tube to provide continuous feedback to the provider. Our previous studies have verified that spectral reflectance can be used to differentiate tracheal and esophageal tissue in ex vivo tissue (Nawn et al., 2016), living, perfused tissue and in human cadavers (Nawn et al., 2019). The model developed in this study represents a crucial step in evaluating spectral reflectance as a viable option in airway management: establishing that tracheal detection could be accomplished during varying levels of oxygen saturation. In that regard, the previously identified tracheal spectral profile (Nawn et al., 2016) is present during all levels of obstruction and allows for differentiation between tracheal and esophageal tissues.

We have hypothesized that this unique profile, made up of two troughs at 543 nm and 578 nm and one peak at 561 nm, is due to the presence of oxygenated hemoglobin which has a similar reflectance pattern (Zijlstra et al., 1991). But given its presence regardless of oxygen saturation status and our previous study which showed that it persists in human cadavers, this raises the possibility that the different profiles may also be intrinsic to the tissues themselves. The trachea and esophagus have three shared layers: the mucosa, submucosa, and adventitia. Where they differ is in their fourth layer, with cartilage in the trachea allowing it to remain rigid and open and a muscularis layer in the esophagus allowing it to contract. Future studies are needed to determine how these structural differences contribute to disparate spectral reflectance profiles.

While the results of this study are promising and identify spectral reflectance as a technology that may be a vital component to future airway management devices, this study is not without limitations. This study supports the notion that spectral reflectance can identify tracheal tissue despite alterations in sO_2_ or pCO_2_, it is important to note that varying levels of oxygen saturation and CO_2_ partial pressure are the only two variables that were examined in this particular study. In patients with airway obstruction, it is likely that they will have other significant comorbidities that may affect tissue reflectance such as the presence of blood, mucus, vomit, or soot in the airway. It will be vital to verify that spectral reflectance remains significantly different between trachea and esophagus in the face of these factors in future studies if this innovative technology is to remain a viable option for tracheal placement verification in trauma patients. Additionally, these studies were undertaken in an animal model, and while swine are a viable model of human physiology and airway anatomy, it is possible that slight differences in tissue makeup and/or structure could alter the spectral reflectance profile. However, given the persistence of the target spectral signature in human cadavers (Nawn et al., 2019), the potential clinical utility remains.

## Conclusion

Overall, the present study validated a large animal model of slow‐ and rapid‐ airway obstruction that results in significant hypoxia and hypercapnia and allowed for the passage of probes into the trachea and esophagus without compromising the obstruction. This model represents an innovative method for future testing of airway device components that may help to improve airway management. Additionally, our data support the ability of spectral reflectance to differentiate between tracheal and esophageal tissue in the face of a clinical condition resulting in decreased oxygen saturation and increased CO_2_ partial pressure. Given the importance of rapid, accurate, and continuous monitoring of the trachea during airway management and ongoing care, incorporating such a tracheal detection component into common airway devices (e.g., ET tube) could aid providers during initial placement of an airway management device and/or during transport.

## DoD Disclaimer

The views expressed in this article are those of the authors and do not reflect the official policy or position of the U.S. Army Medical Department, Department of the Army, DoD, or the U.S. Government.

## Conflict of Interest

None declared.
